# Activation of Tissue Remodeling Precedes Obliterative Bronchiolitis in Lung Transplant Recipients

**DOI:** 10.4137/bmi.s686

**Published:** 2008-06-06

**Authors:** Allan M. Ramirez, David R. Nunley, Mauricio Rojas, Jesse Roman

**Affiliations:** 1 McKelvey Center for Lung Transplantation and Pulmonary Vascular Diseases; 2 Division of Pulmonary, Allergy and Critical Care Medicine, Emory University School of Medicine, Atlanta; 3 Division of Pulmonary, Critical Care and Sleep Medicine, The Ohio State University School of Medicine, Columbus; 4 Atlanta Veterans Affairs Medical Center

**Keywords:** tissue remodeling, transforming growth factor-beta, fibronectin, matrix metalloproteinase, lung transplant

## Abstract

Obliterative bronchiolitis (OB) and Bronchiolitis Obliterans Syndrome (BOS) are frequent complications in the lung transplant recipient, and are the leading cause of mortality after transplantation. The mechanisms responsible for OB remain elusive, but inflammatory and tissue remodeling responses are implicated. We hypothesized that alterations in markers of tissue remodeling in BALF of lung transplant recipients could predict development of OB. To test this, we identified 13 lung transplant recipients who developed both BOS and histologic OB (OB group) at median post-operative day (POD) 485 (range 73–2070). Bronchoalveolar lavage fluid (BALF) was obtained at median POD 387 (range 45–2205), which preceded the onset of OB and BOS by a median of 140 days (range 60–365). As a control, BALF was also obtained from a group of 21 stable recipients without OB (non-OB group) at median POD 335 (range 270–395). BALF was examined for gelatinolytic activity, fibronectin gene transcription, and transforming growth factor-β1 (TGF-β1) expression. Gelatin zymography of BALF from the OB group showed increased matrix metalloproteinase-9 (MMP-9) activity over that of the non-OB group (p < 0.005). Similarly, BALF from the OB group induced greater fibronectin expression in fibroblasts compared to the non-OB group (p < 0.03). The induction of fibronectin also correlated with the amount of TGF-β1 protein in BALF (r = 0.71) from the OB group. We conclude that activation of tissue remodeling precedes the onset of OB, and analysis of gelatinolytic and/or fibronectin-inducing activity in BALF can serve as an early, pre-clinical marker for OB.

## Introduction

Over the past twenty years, lung transplantation (LTX) has evolved into a viable treatment option for patients with end-stage lung disease resulting from a variety of pulmonary insults. However, allograft rejection with the development of obliterative bronchiolitis (OB) remains the single greatest impediment to long-term survival following lung transplantation (Estenne and Hertz, 2002). The diagnosis of OB, which complicates nearly 60% of lung allografts, is made histologically with the demonstration of obstructing intraluminal plugs of fibromyxoid tissue in terminal bronchioles. This entity becomes more prevalent with an increasing interval since the transplant procedure (Sundaresan et al. 1995). Often accompanied by recurrent airway infections (Paradis et al. 1993), if unabated, OB ultimately results in progressive ventilatory insufficiency and death. The clinical correlate of OB is termed the bronchiolitis obliterans syndrome (BOS) and is manifested in the allograft recipient by a reduction in forced expiratory volume in 1 second (FEV_1_) of at least 20% in the absence of other identifiable causes (Cooper et al. 1993).

Obliterative bronchiolitis may have an inhomogeneous distribution in the allograft. Consequently, the sensitivity of transbronchial lung biopsy for detecting OB approaches only 15%–40%, even with a high clinical suspicion (Kramer et al. 1993; Ladowski et al. 1993). Once identified, OB typically responds poorly to augmented immunosuppression. Because the histologic confirmation of OB may be difficult, alternative means have been investigated in an attempt to identify surrogate markers for OB. Perhaps, the identification of such markers may be more useful should they accurately predict the later development of OB at a stage when directed therapy may be more successful.

Most studies on the pathogenesis of OB have concentrated on the role of cellular and/or humoral allogeneic immune responses after LTX (Henke et al. 1999; Leonard et al. 2002). Other studies point to inflammatory mediators as important players in the pathogenesis of OB (De Andrade et al. 2000; DiGiovine et al. 1996; Reynaud-Gaubert et al. 2002). More recently, attention has been given to factors that control the *tissue remodeling* or *fibro-proliferative* response of the lung to injury. This tissue remodeling response is responsible for the accumulation of fibroblasts and the excess deposition of connective tissue matrices within the airway lumen causing the progressive airflow limitation characteristic of OB (Roman, 1998). Little is known about the factors that trigger and maintain this tissue remodeling response in LTX recipients.

From our unpublished observations, we found evidence for differential tissue remodeling activity in allografts of lung transplant recipients. Specifically, gelatinolytic activity, a marker of connective tissue degrading capability, was increased in the bronchoalveolar lavage fluid of all recipients during the first 3 months following transplantation. This activity, however, remained elevated only in those recipients who met criteria for bronchiolitis obliterans syndrome. Based on these preliminary data, we subsequently analyzed lung lavage fluid from 34 lung transplant recipients whose allograft function was clearly defined with respect to the presence of OB and BOS. Lavage fluid obtained before either the clinical or histologic onset of OB/BOS was analyzed for three markers of tissue remodeling; namely, gelatinolytic activity, induction of fibronectin expression in fibroblasts, and the expression of the profibrotic growth factor, transforming growth factor–β1 (TGF-β1). In addition, we examined the lavage fluid for cytokine markers of inflammation as potential precursors to OB.

## Materials and Methods

### Study subjects

Study subjects included lung transplant recipients who were followed at the University of Pittsburgh Medical Center, Pittsburgh, PA between January 1, 1995 and December 31, 2000. Recipients, aged from 18 to 65 years old, received a standard maintenance immunosuppressive regimen which consisted of a calcineurin inhibitor, either cyclosporine or tacrolimus. Cyclosporine doses were adjusted to maintain whole blood trough levels of between 200 and 250 ng/ml, while tacrolimus doses were adjusted to maintain whole blood trough levels between 10 and 20 ng/ml. In addition to the calcineurin inhibitor, the maintenance immunosuppressive regimen also consisted of azathioprine (0.5 to 1 mg/kg per day), to maintain a white blood cell count above 5,000/mm^3^; and prednisone, which was slowly tapered to a nadir of zero to 5 mg per day. Episodes of acute cellular allograft rejection were treated with intravenous methyl-prednisolone 1 gram per day for 3 consecutive days. The diagnoses of OB and BOS were established according to the criteria of the International Society of Heart and Lung Transplantation (Cooper et al. 1993).

### Bronchoalveolar lavage fluid collection

After obtaining informed consent, bronchoalveolar lavage fluid (BALF) was collected, and a database consisting of contemporaneous clinical information was compiled. Samples were obtained during routine surveillance of lung allografts according to the following schedule: every 3 months during the first postoperative year, every 4 months during the second postoperative year, every 6 months during the third postoperative year, and once each year beginning with the fourth postoperative year. Additionally, data and BALF were collected when bronchoscopy was performed for any change in the recipient’s clinical and/or functional status. Bronchoalveolar lavage was performed by instilling a total of 200cc of normal saline into a subsegment of the lingula or the right middle lobe. The database and BALF samples were transferred to and analyzed at Emory University (by D. N.). The study was approved by the institutional review boards of the University of Pittsburgh and Emory University.

### Protein determination

To ensure valid comparisons of BALF analyses, total protein concentration was determined using the Protein Assay Dye Reagent according to manufacturer instructions (Bio-Rad, Hercules, CA). Aliquots of BALF containing equivalent amounts of protein were subsequently used for the testing described below.

### Gelatin zymography

BALF was fractionated by electrophoresis in 8% SDS-polyacrylamide gels containing 0.3 mg/ml of gelatin (Sigma, St. Louis, MO) under nonreducing conditions (Rivera-Marrero et al. 2000). Prestained molecular weight markers (Amersham Biosciences, Piscataway, NJ) along with a purified matrix metalloproteinase (MMP)-9 standard (EMD Biosciences, La Jolla, CA) were included to estimate the molecular weight of the gelatinolytic bands. The gels were washed with 2.5% Triton X-100, incubated overnight at 37 °C in incubation buffer (50mM Tris pH 7.5, 5 mM CaCl_2_), stained with 0.25% Coomassie blue R-250 (Sigma Chemical Company, St. Louis, MO) in water/methanol/acetic acid (45:45:10), and destained with 50% methanol in water. Gelatinolytic activities, detected as a transparent band in the remaining blue-stained gel, were analyzed by densitometry.

### Cell culture and fibronectin gene expression

To study the ability of BALF to stimulate fibronectin expression, the full length fibronectin promoter connected to a luciferase reporter vector, pFN(1.2 kb)LUC, was introduced into murine NIH3T3 fibroblasts from the American Type Culture Collection (CRL #1658; Rockville, MD) via electroporation to create stable transfectants as previously described (Michaelson et al. 2002). Fibroblasts were maintained at 37 °C in a 5% CO_2_ atmosphere in Dulbecco’s modified Eagle’s medium (Mediatech, Herndon, VA) supplemented with 10% heat-inactivated fetal bovine serum (FBS) and 1% antibiotic-antimycotic (100 U/ml penicillin G sodium, 100 U/ml streptomycin sulfate, and 0.25 g/ml amphotericin B). The cells were harvested by trypsinization with 2.5X trypsin and 5.3 mM EDTA (Sigma), washed with PBS, plated at 1.5 × 10^5^ cells/ml in 6 well tissue culture dishes, and grown to near confluence. Following serum starvation for 24 hours and incubation with 300μl aliquots of BALF with equal protein content in 700 μl serum-free media (Mediatech) for 72 hours, fibroblasts were harvested by cell scraper, washed with PBS, resuspended in 100 μl of cell lysis buffer (Promega), and sonicated. Light intensity of a 10-μl aliquot of cell lysate was tested by adding 50 μl of luciferase assay reagent (Promega, Madison, WI) and measured using a ThermoLabsystems Luminoskan Ascent microtiter plate luminometer. Experiments were performed in duplicate and repeated, with results recorded as luciferase units, normalized to total protein content.

### Enzyme linked immunosorbent assay

TGF-β1 in BALF was assayed in duplicate by sandwich enzyme-linked immunosorbent assay using the TGF-β1 Emax^®^ ImmunoAssay System (Promega, Madison, WI) according to manufacturer protocol. Briefly, 96-well flat-bottom plates (Nalge Nunc International, Rochester, NY) were coated with TGF-β1 coating monoclonal antibody overnight at 4 °C, blocked with Block Buffer, and incubated with BALF for 90 minutes. Plates were then incubated with a second anti-TGF-β1 polyclonal antibody, washed with tris-buffered saline with 0.05% Tween-20 (Sigma, St. Louis, MO), and incubated with TGF-β1 HRP Conjugate. A 3-3′,5,5′-tetramethylbenzidine/peroxidase reagent solution was added to develop color. The reaction was terminated with 1 N HCl and the optical density was measured at 450 nm in an AD 340 Plate Reader (Beckman Coulter, Fullerton, CA). A standard curve was generated using serial dilutions of known amounts of TGF-β1.

### Detection of cytokine protein in BALF

A Multiplex Bead Immunoassay, that uses micro-spheres labeled with unique fluorophores in a single well of a filter bottomed ELISA plate, was used to analyze cytokine protein levels in BALF samples. A 10 plex-kit (Biosource, Camirillo, CA) was used that allowed for simultaneous detection of: IL-1β, IL-2, IL-4, IL-5, IL-6, IL-10, IL-12, IFN-γ, GM-CSF and TNF-α. Well filters were pre-washed and 50 μl of 1:1 diluted sample are applied to each well. Then, antibody-coated beads specific for the ten different cytokines were added to the wells and incubated for 2 h at room temperature in a shaker. After incubation, the plate was washed twice using a Manifold washer. Biotinylated antibodies against the ten cytokines were added to the reaction and incubated during an hour. Afterwards, the cytokine-antibody complexes were detected by adding streptavidin coupled to phycoerythrin. The number of positive complexes was determined by reading each sample in a Luminex XYP platform. Data obtained in duplicates were analyzed using a MasterPlex 1.2 from Mai-Rabio, and data related to concentration was expressed in pg/ml.

### Statistical analysis

Data are expressed as mean ± SD. Means between study groups were compared using the two-tailed student’s t-test with unequal variance. Logistic regression analysis was utilized to identify a relationship between independent and dependent variables. A “p” value less than 0.05 was considered significant. Each BALF sample was conducted in duplicate with consistent results.

## Results

Utilizing a database which consisted of 260 LTX recipients, 13 recipients who developed histologic OB and also met criteria for BOS (OB group) were identified, as were 21 recipients who never developed OB or BOS (non-OB group). The non-OB group consisted of all transplant recipients during the study period who: 1) never had histologic OB on lung biopsy, 2) did not fulfill FEV1 criteria for BOS, 3) were free of pulmonary infection (as evidenced by negative BALF cultures) at the designated study time, and 4) had no evidence of acute allograft rejection (by biopsy) at the designated study time. As described in Table 1, recipients underwent single lung (n = 18), or bilateral lung (n = 16) transplantation for end-stage lung disease resulting from emphysema (n = 10), pulmonary fibrosis (n = 8), cystic fibrosis (n = 4), or pulmonary hypertension (n = 12).

In the OB/BOS group, a cross-sectional analysis was performed on BALF obtained during the most proximate bronchoscopy *prior* to the diagnosis of OB/BOS, at a time when subjects were clinically-well and free of OB, BOS, acute rejection, and infection. This occurred at median postop day 387 (range 45–2205) and corresponded to a median of 140 days (range 60–365) before the onset of OB/BOS. From among the serial lavage fluid specimens of the non-OB group, the BALF selected for cross-sectional study was sampled at median post-op day 335 (range 270–395) when: 1) subjects had no clinical evidence of allograft disease and 2) number of days post-op most closely matched that in the OB/BOS group.

### Gelatinolytic activity in BALF of lung transplant recipients

**Figure 1:**
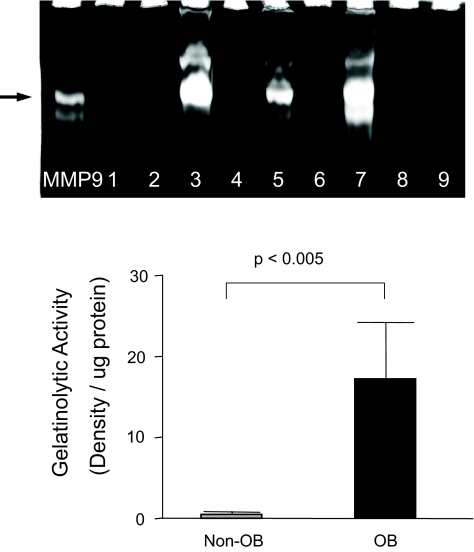
Gelatinolytic activity is increased in the BALF of lung transplant recipients who later develop OB Gelatin zymography performed on BALF specimens from lung transplant recipients revealed gelatinolytic activity at ~92 kD. Depicted is a representative blot illustrating MMP-9 activity of BALF from recipients who later developed OB (lanes 3, 5, 7) and those who did not (lanes 1, 2, 4, 6, 8, 9). Densitometric analysis of the gelatin zymograms showed a nearly 20-fold increase in gelatinolytic activity in BALF from the OB group compared to non-OB group (p < 0.005).

The study of BALF by gelatin zymography revealed bands of degradation near 92 kD, corresponding to the purified MMP-9 control. Compared to the non-OB group, densitometric analysis of the zymogram of BALF samples from the OB group showed a nearly 20-fold increase in gelatinolytic activity per microgram of protein (p < 0.005) (Fig. 1).

### Fibronectin gene transcription and TGF-β1 expression

**Figure 2:**
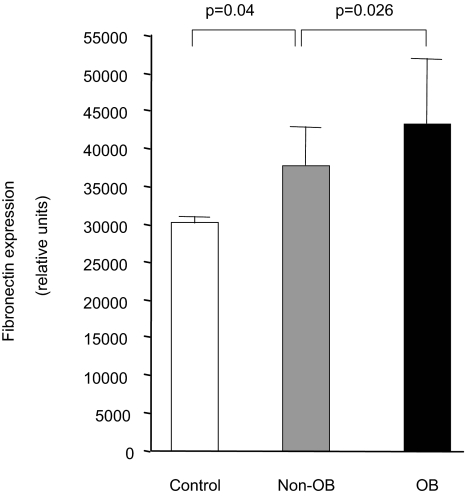
Fibroblast induction of fibronectin by BALF is greater from recipients who develop OB Fibroblasts transfected with a fibronectin promoter-luciferase reporter construct were cultured with BALF for 72 hours. Fibronectin gene transcription was higher in fibroblasts exposed to BALF from the OB groups when compared to that from the non-OB group.

Experiments were also conducted to ascertain if BALF contained soluble factors that could stimulate the production of extracellular matrix components like fibronectin, a matrix glycoprotein highly expressed in allograft rejection (Hirabayashi et al. 1996). To this end, NIH3T3 fibroblasts permanently transfected with the human fibronectin promoter connected to the luciferase reporter gene, pFN(1.2 kb)LUC, were cultured in the presence of BALF and processed for luciferase activity. As demonstrated in Figure 2, fibronectin promoter activity was present in control fibroblasts exposed to saline. The fibronectin promoter activity was significantly greater after 72 hours in fibroblasts incubated with BALF from transplant recipients who later develop OB when compared to cells exposed to BALF from the non-OB group (p = 0.026).

**Figure 3:**
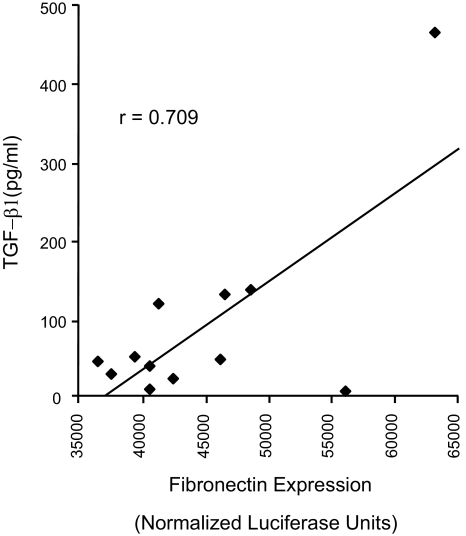
TGF-β1 correlates with fibronectin expression in BALF TGF-β1 in BALF was determined by enzyme-linked immunoabsorbent assay. A strong correlation was noted between TGF-β1 levels and fibronectin expression in BALF from the OB group (r = 0.709).

The pro-fibrotic cytokine TGF-β1 has been implicated in the pathogenesis of OB and is a powerful regulator of fibronectin expression (Blobe et al. 2000). Consequently, the association of TGF-β1 with fibronectin induction in OB was also examined. TGF-β1 protein levels were measured in BALF from the OB group by ELISA testing and a strong correlation between TGF-β1 protein levels and fibronectin gene transcription was found (r = 0.71) (Fig. 3). However, TGFβ1 levels, by themselves, were not found to be predictive of OB in this cohort (data not shown).

### Cytokine levels in BALF

**Figure 4:**
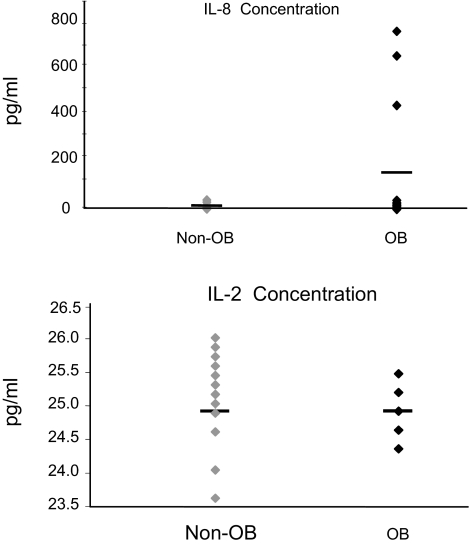
Cytokine levels in BALF of OB and non-OB samples The analysis of cytokine levels in the BALF of individual recipients revealed that 3 of 13 recipients in the OB group had markedly elevated levels of IL-8 in their BALF. IL-1β, IL-6, and TNF-α had a similar pattern in these recipients. In contrast, IL-2 levels were narrowly distributed about the mean in both groups. IL-4 and IL-10 expression were likewise similar.

A multiplexed, fluorescent microsphere-based immunoassay was performed using the Luminex^®^ platform to quantify multiple cytokines simultaneously in each single sample. Microspheres were customized to detect the cytokines listed in Table 2. Of the ones measured, no differences in cytokine levels could be found in the BALF from the OB and non-OB groups. A trend toward statistical significance was noted for interleukin-8 (IL-8) (p = 0.08). Interferon-γ, granulocyte-macrophage colony stimulating factor (GM-CSF), and interleukin-5 could not be detected above background levels. Cytokine levels for each recipient were also individually analyzed. Representative graphs are depicted in Figure 4. Although no difference was seen in the amounts of IL-8 (shown), IL-6, IL-1β, and tumor necrosis factor-α, the same three recipients in the OB group had markedly elevated amounts of these cytokines in their BALF. In contrast, the level of IL-2 (shown), IL-4, and IL-10 in both groups were tightly clustered around the mean.

## Discussion

The identification of markers capable of predicting the development of OB in lung transplant recipients would have useful prognostic value, and would serve to identify recipients who might benefit from early intervention. Unfortunately, other than clinical risk factors such as repeated infections and early rejection, effective predictive factors for OB post lung transplantation are not available (Estenne et al. 2002). Most studies in this area have focused on the inflammatory cascade. For instance, BALF granulocytic markers, such as eosinophilia/neutrophilia, eosinophil cationic protein, and interleukin-6 (Riise et al. 1999) (Scholma et al. 2000), may herald the development of chronic allograft dysfunction, though, interestingly, they also represent biomarkers for acute cellular rejection, a well-established clinical risk factor for OB. Here, we focus on markers of tissue remodeling, another process known to be activated during tissue injury. In the lung, like other organs, activation of tissue remodeling is characterized by alterations in the expression or degradation of connective tissue matrices. Tissue remodeling is activated in lung rejection, and the pathologic features of OB reflect its importance.

First described in the mid-1980’s (Burke et al. 1984), histologic specimens obtained from affected recipients showed intraluminal granulation tissue and dense submucosal eosinophilic plaques. Obliteration of the terminal airways by these lesions results in progressive airflow obstruction. The observation that granulation tissue is deposited in the airways of recipients with OB has led to the suspicion that this finding represents a reparative response to some, as of yet, unidentified injurious process. This initiating process is suspected of resulting in connective tissue remodeling thus culminating in the obliteration of terminal airways. Obliterative bronchiolitis is characterized histologically, in part, by the deposition of collagen matrix within the submucosa of terminal bronchioles. In contrast to normally occurring Type I collagen of healthy airways, porcine models of OB have demonstrated a predominance of Type III procollagen mRNA in airway fibroblasts suggesting that fibroblast stimulation results in the deposition of collagen matrix (Alho et al. 2001). While several profibrotic mediators are suspected, the stimulus to this production of new matrix is unclear. This has prompted a search for profibrotic mediators in the airway of lung transplant recipients (Bergmann et al. 1998; Charpin et al. 1998).

In preliminary work generated from the analysis of BALF samples obtained at random from lung transplant recipients, we found that the early post-surgical period (<3 months) is characterized by increased levels of gelatinolytic activity. This activity tended to decrease after this period, but remained elevated in recipients with OB when compared to those who remained stable (Boles and Roman, unpublished observations). These data suggested that gelatinolytic activity could serve as a predictor of OB development. However, the retrospective nature of this analysis did not allow for a careful characterization of the recipients, or for a thorough analysis of the data against parameters such as time of diagnosis of OB, among other factors.

To address these deficiencies, we engaged in an analysis of samples collected from 260 recipients of lung transplantation. Within this group, we identified a well-characterized subset of recipients who were subjected to bronchoscopy for surveillance purposes prior to the diagnosis of OB (n = 13). Sequentially collected BALF samples were available from all of these recipients as well as from recipients who remained stable (n = 21). The BALF samples obtained from these recipients prior to the diagnosis of OB represent the experimental material for this study.

We found increased gelatinolytic activity in the BALF of recipients who later developed OB. These data have important implications for the following reasons. First, the data were obtained using gelatin zymography, a simple yet effective method of detecting gelatinolytic activity that could be easily established in clinical laboratories. Second, the early detection of gelatinolytic activity might reflect important pathophysiological mechanisms that should be investigated. The activity detected was located at 92 kD consistent with active MMP-9. MMP-9 is a zinc-dependent endopeptidase that is secreted inactive from macrophages and epithelial cells, among other cells (Birkedal-Hansen et al. 1993). Extracellular MMP-9 can be activated via proteolysis by other proteases including plasmin and other MMPs. Activated MMP-9 is capable of degrading type IV collagen, fibronectin, elastin and other connective tissue matrices (Nagase and Woessner, 1999). It also can affect cellular chemotaxis and the activation of soluble growth factors such as TGF-β1. Rather than measuring MMP-9 levels, we examined a functional property of MMP-9, i.e. gelatinase activity, as a marker of tissue remodeling since it not only incorporates the expression of active MMP-9, but reflects the balance of MMP-9 activity and its inhibitor, TIMP-1.

The detection of increased levels MMP-9 in acute lung injury (Delclaux et al. 1997; Ricou et al. 1996), idiopathic pulmonary fibrosis (Suga et al. 2000), cystic fibrosis (Delacourt et al. 1995), bronchiectasis (Sepper et al. 1994), and other lung disorders characterizes MMP-9 as a sensitive marker of activation of tissue remodeling after lung injury, but it is unlikely to serve as a indicator of a specific injurious agent or entity in the pathophysiology of many acute and chronic forms of disease. Among lung transplant recipients, MMP-9 has been found to be upregulated in BALF from those with BOS (Hubner et al. 2005; Taghavi et al. 2005). In our cohort of patients, we found that the gelatinolytic activity of MMP-9 in BALF was a strong predictor of both pulmonary function limitation and histologic airway fibrosis, suggesting a pathophysiological role for this protease linking the matrix remodeling to the airflow obstruction in OB. Indeed, causality for MMP-9 in the pathogenesis of OB has been proposed in studies using an animal model of chronic allograft rejection with MMP-9- deficient mice (Fernandez et al. 2005).

We also found that the BALF obtained from recipients in the OB group stimulated the expression of fibronectin in cultured fibroblasts. Fibronectin is a matrix glycoprotein highly expressed in acute and chronic forms of lung injury; it, too, is considered an early marker of disease. Fibronectin is produced by fibroblasts, epithelial cells, and alveolar macrophages and the intact molecule or its fragments modulate cellular processes such as adhesion, migration/chemotaxis, proliferation, and in monocytic cells, cytokine expression (Roman, 1998; Roman, 1996; Roman et al. 2000). The observed increase in its stimulation in the OB group, suggests that, like gelatinolytic activity, this molecule could serve as an early marker of injury that is likely to progress to OB.

Fibronectin expression is stimulated by a number of factors such as TGF-β1. The correlation between BALF levels of TGF-β1 protein and fibronectin stimulation in our assay suggest a causal role for TGF-β1. In other work, we demonstrate that interruption of TGF-β1 signaling through its intracellular signal transducer, Smad3, ameliorates experimental OB and results in decreased expression of fibronectin (Ramirez et al. 2004).

In addition to the above, we evaluated the BALF samples for a number of cytokines. However, no statistically significant differences were observed between the OB and non-OB groups. Only IL-8 showed a trend towards significance, and this is consistent with data from others showing upregulation of IL-8 during episodes of ischemia-reperfusion and active OB (De Perrot et al. 2002; DiGiovine et al. 1996). Although the number of patients tested was relatively small, our data, like that of DiGiovine et al.(DiGiovine et al. 1996), suggest that IL-8 and other cytokine measurements in BALF are not highly predictive markers of future development of OB. Indeed, consistently increased levels of IL-8 among cystic fibrosis recipients were not found to be associated with any allograft rejection (Dosanjh et al. 1998).

Together, our data suggest that OB in lung transplant recipients is preceded by the activation of tissue remodeling which can be detected in BALF as reflected by increased gelatinolytic activity and BALF ability to stimulate fibronectin expression in fibroblasts. Further investigations will be needed to determine the true predictive value of these assays in lung transplant recipients.

## Figures and Tables

**Table 1:** Characteristics of study subjects.

Parameter	Non-OB	OB/BOS
Lung transplant diagnosis (*n*)	21	13
Emphysema	6	4
Pulmonary fibrosis		
Idiopathic	0	3
Collagen vascular disease-associated	4	1
Cystic fibrosis	2	2
Pulmonary hypertension		
Idiopathic PAH	2	1
Eisenmenger’s syndrome	7	2
Bilateral lung transplant	10	6
Post-op days to OB/BOS diagnosis*	N/A	485 (73–2070)
Post-op day BALF obtained*	335 (270–395)	387 (45–2205)
Days BALF collected before OB/BOS*	N/A	140 (60–365)

**Abbreviations:** OB: obliterative bronchiolitis; BOS: bronchiolitis obliterans syndrome; BALF: bronchoalveolar lavage fluid.

* Values expressed as median (range).

**Table 2:** Concentration of cytokines in bronchoalveolar lavage fluid.

Cytokine	Non-OB	OB/BOS	p value
GM-CSF	ND	ND	–
Interferon-γ	ND	ND	–
Interleukin-1β	20.32 ± 1.60	22.67 ± 6.76	0.24
Interleukin-2	25.07 ± 0.56	24.85 ± 0.16	0.14
Interleukin-4	30.11 ± 0.53	30.00 ± 0.50	0.53
Interleukin-5	ND	ND	–
Interleukin-6	13.83 ± 0.55	35.26 ± 51.69	0.16
Interleukin-8	9.46 ± 8.41	159.65 ± 281.53	0.08
Interleukin-10	16.67 ± 0.07	16.67 ±0.08	0.99
TNF-α	12.09 ± 0.66	14.19 ± 5.99	0.23

**Abbreviations:** OB: obliterative bronchiolitis; BOS: bronchiolitis obliterans syndrome; ND: not detectable; GM-CS: granulocyte-macrophage colony stimulating factor; TNF-α: tumor necrosis factor-alpha. Data are depicted as mean (pg/ml) ± SD.
